# Exaggerated Trait Allometry, Compensation and Trade-Offs in the New Zealand Giraffe Weevil (*Lasiorhynchus barbicornis*)

**DOI:** 10.1371/journal.pone.0082467

**Published:** 2013-11-27

**Authors:** Christina. J. Painting, Gregory I. Holwell

**Affiliations:** School of Biological Sciences, University of Auckland, Auckland, New Zealand; Estacion Experimental de Zonas Áridas (CSIC), Spain

## Abstract

Sexual selection has driven the evolution of exaggerated traits among diverse animal taxa. The production of exaggerated traits can come at a cost to other traits through trade-offs when resources allocated to trait development are limited. Alternatively some traits can be selected for in parallel to support or compensate for the cost of bearing the exaggerated trait. Male giraffe weevils (*Lasiorhynchus barbicornis*) display an extremely elongated rostrum used as a weapon during contests for mates. Here we characterise the scaling relationship between rostrum and body size and show that males have a steep positive allometry, but that the slope is non-linear due to a relative reduction in rostrum length for the largest males, suggesting a limitation in resource allocation or a diminishing requirement for large males to invest increasingly into larger rostra. We also measured testes, wings, antennae, fore- and hind-tibia size and found no evidence of a trade-off between these traits and rostrum length when comparing phenotypic correlations. However, the relative length of wings, antennae, fore- and hind-tibia all increased with relative rostrum length suggesting these traits may be under correlational selection. Increased investment in wing and leg length is therefore likely to compensate for the costs of flying with, and wielding the exaggerated rostrum of larger male giraffe weevils. These results provide a first step in identifying the potential for trait compensation and trades-offs, but are phenotypic correlations only and should be interpreted with care in the absence of breeding experiments.

## Introduction

Competition between males for mates has led to the evolution of exaggerated traits through sexual selection [1,2]. Exaggerated traits can be used as either weapons to increase success in direct conflict with other males, or as ornaments subject to female choice. Males possessing the largest or most elaborate traits are often demonstrated to benefit from higher mating success than their counterparts [1]. 

In many species sexual selection for an exaggerated trait has resulted in a steep positive allometric relationship with body size, such that the trait gets disproportionately larger with increasing body size [3], but see 4. Exaggerated trait scaling relationships are often best described by a straight line after log transformation of body and weapon size, but in many species the relationship is non-linear and is better described as being curved, sigmoidal or discontinuous [5]. Many species exhibit dimorphism in male weapon size as a discontinuity in the scaling relationship between body and weapon size. Weapon dimorphism has been traditionally associated with a developmental threshold, such that only males above a certain body size are reprogrammed to display the trait, and below the threshold the trait will be rudimentary or absent [6]. However, recent analyses have shown that these thresholds are not always straightforward, and that some model species such as the scarabaeid beetle *Onthophagus taurus* show a continuous relationship between weapon and body size and do not show evidence for a reprogramming event between small hornless (minor) and large horned (major) males, supporting instead a non-linear model of exponential horn growth followed by a growth constraint in the largest males due to competition between body parts during resource allocation [7]. Furthermore, the weapon dimorphisms of some species are not highlighted by a bimodal frequency distribution, and are only detected by analysing scaling relationships using non-linear techniques [5]. In many cases the resulting dimorphism between small and large males is coupled with important differences in behaviour between the two morphs, such as alternative mating tactics [8,9]. These tactics are generally divided at a threshold between minor and major males, and in many cases minors adopt sneaking tactics while majors rely on aggressive mate guarding tactics [10-12]. 

The development of exaggerated secondary sexual traits such as horns and enlarged mandibles can come at the cost of investment in other characters due to the trade-off for resources prior to development into adults [13]. The demand placed on the development of the adult by the exaggerated trait can result in an overall reduction in body size, or smaller individual traits such as wings and eyes. Alternatively some adult body structures may be under genetic correlational selection for their role in supporting the exaggerated trait, such that the resulting phenotype of the body part correlates with the phenotype of the trait in question [14]. Compensation can also occur where the potential reduction in performance (e.g. locomotion) due to bearing a large exaggerated trait results in the correlated increase in another trait (e.g. wing size). These correlated traits may physically support the growth of the exaggerated trait, play a role in its use, or compensate for any negative impact on another aspect of the individuals performance. For example, the exaggerated eye-span of large male stalk-eyed flies should cause a major disadvantage in flight performance due to the cost of bearing this trait; however studies of these species have shown a surprising lack of male disadvantage in flight, likely due to correlational selection on increased wing size in relation to the increased investment in eye span [15-17]. Overall, individuals which have large exaggerated traits are expected to possess larger supporting or compensatory structures which are selected alongside the exaggerated trait, while also being subject to trade-offs with other body parts. 

Resource investment into the development of each trait is particularly important in holometabolous insect species because development during metamorphosis takes place in a closed system [14]. During larval development, clusters of cells form to make up imaginal structures (or imaginal discs) that will become adult appendages after metamorphosis. Each group of cells is separate such that, for example, the cluster that will form the left leg is separate from that which will form the right leg [18]. Although these cells are set aside early in larval growth during embryogenesis, most of the growth of imaginal discs occurs after the larva has stopped feeding, prior to metamorphosis into the pupal stage [19,20]. This has implications for development because imaginal discs must share resources that have been previously acquired. The final size of each imaginal disc is a function of body size, complicated by the fact that they grow in a closed system where disc growth comes at the expense of other parts of the body and overall size [20]. This can result in morphological trade-offs where the development of an exaggerated trait (e.g. horns) can result in other structures (e.g. eyes, genitalia) growing disproportionately smaller [13]. 

The expression of exaggerated traits can also be limited such that after a certain size relative to the body, further exaggeration of the trait would result in problems with the development of crucial parts of the rest of the body [21]. This is observed as a curvature or asymptote in the slope when plotting weapon size against body size, and shows that the largest males in the species actually decrease their relative investment in weapon size [22]. Although this decreasing slope at the upper size limit of males has been described in some studies, it has been relatively overlooked until recently when Knell et al. [21] conducted a comparative analysis using lucanid beetles to show that the largest males of a species had decreasing investment in weapon size, and that across the family species with the largest mandibles had the greatest decrease of slope for large males. The closed system for development of holometabolous species as described above may explain these asymptotic allometries because resources must be shared between traits, thereby resulting in competition between traits during growth and development. Individuals that invest heavily into an exaggerated trait could therefore deplete any available resources for growth resulting in a physiological constraint in trait size [21]. 

**Figure 1:**
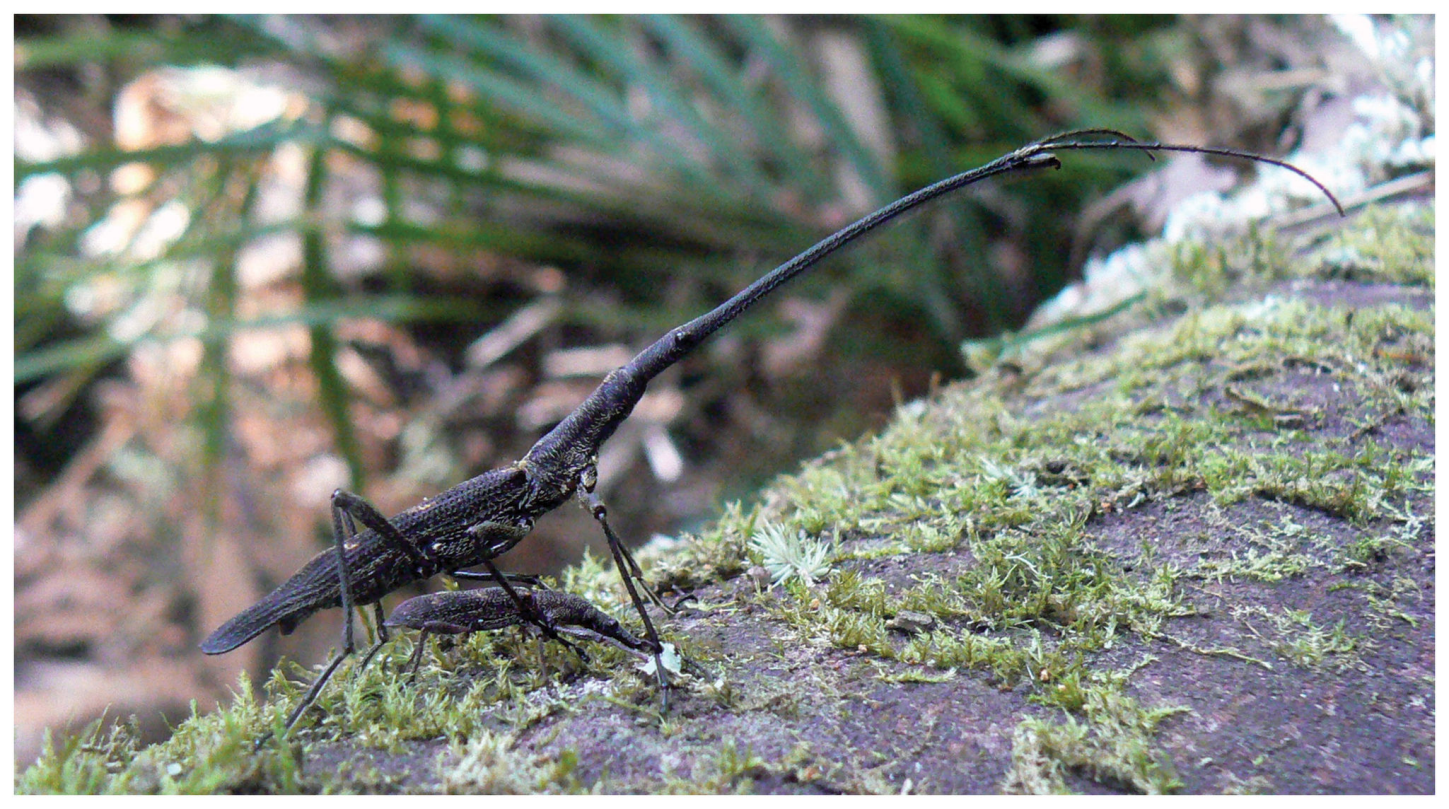
A large male giraffe weevil guards a drilling female. The extreme sexual dimorphism in this species is highlighted here, and is mostly due to the elongation of the male rostrum.

The New Zealand giraffe weevil *Lasiorhynchus barbicornis* (Fabricius) (Coleoptera) is the longest brentid weevil in the world, mostly owing to the extremely elongated rostrum (extension of head) displayed by adult males (Figure 1). Although both sexes show high size variation, females are generally the smaller sex (12 - 50 mm in length), with males varying six-fold in size from 15 - 90 mm in length (CJ Painting unpublished data) [23]. Giraffe weevils are an endemic wood-boring species in New Zealand, found in native forest in association with dead or dying trees. Adult weevils emerge and aggregate in summer months on tree trunks or branches where females will drill the tree surface to prepare a hole suitable for laying an egg [24]. During this time males will compete fiercely for access to females for copulation, using their greatly elongated rostrum and enlarged mandibles to push, bite, pull and grapple other males from the female, occasionally throwing their opponent off the tree (CJ Painting personal observations) [24]. The use of the rostrum by males during these contests highlights its importance as a weapon, and suggests that it is likely to be under direct sexual selection, however the scaling relationship between rostrum and body size is currently undescribed. 

This study (1) characterises the shape and slope of the scaling relationship between rostrum length and body size of male and female giraffe weevils (2), investigates the presence of an asymptote in weapon allometry (3), checks for discontinuities in the scaling relationship between weapon and body size as possible indicators of male dimorphism in weapon size and the potential for the presence of alternative mating tactics and (4) investigates the potential for trade-offs and/or compensation between weapon size and that of testis, wing, antennae, and leg size by investigating phenotypic correlations. A description of the scaling relationship of rostrum size for this species is important as a morphological framework from which to base future studies on the behavioural importance of this trait as a weapon in males. A decreasing allometry for the largest males can suggest an increasing cost in weapon production due to a limit in resource allocation. Alternatively, the relative benefits of increasing investment in rostrum length could diminish as males approach the largest size in the population. If weapon size determines fighting success, a very large male already has the competitive advantage over the majority of other males in the population, and therefore would be under less selective pressure to invest further in rostrum size. Given the extreme length of large males and the heavy investment into rostrum length, any trade-off or compensation in the relative size of other traits can help us to understand the cost of bearing such a greatly exaggerated trait. A previous study found that the strongest phenotypic trade-offs were between the weapon and the nearest developing trait (e.g. eyes, wings or antenna), which is hypothesised to be due to local competition between traits for a limited pool of resources [13]. The antennae of *L. barbicornis* grow directly from the rostrum, and we therefore predict that antenna size will show a phenotypic trade-off with rostrum size. Furthermore, we predict a trade-off between testis size and rostrum length because animals are expected to have a limited amount of resources for reproduction, which should be divided between the ability to gain access to females (e.g. increased weapon size) and fertilisation through increased sperm production (testis size) [25]. We assume that there is a biomechanical cost to bearing weaponry, and that therefore wings and legs will be increased in size to assist with flying ability and wielding of the weapon. 

## Materials and Methods

### Field sampling and morphological measurements

Measurements of 987 male and 653 female adult *L. barbicornis* were taken to characterise the scaling relationship between body and weapon size from a wild population at Matuku Reserve (36°51’55.205”S, 174° 28’19.262”E) in West Auckland, New Zealand. We conducted weekly trips to the reserve between 4 November 2011 and 26 March 2012 to take regular snapshots of the giraffe weevil population. Given that body size and scaling relationships for some species can vary significantly during a season [26-28], taking representative measurements from the whole season provides a baseline description of the scaling relationship from which to compare in the future. Weevils were found by searching on 16 dying or partially dying, standing karaka (*Corynocarpus laevigatus*) trees which we had previously determined to host aggregations of adult *L. barbicornis*. Searching was conducted systematically along the trunk and branches of each tree until all weevils present were located. The weevils were removed, measured, then marked with a unique colour combination (Queen Bee Marking Paints, Lega, Italy) to ensure they were only ever measured once during the season, and then released back onto the trees. Measurements were made using Rok digital calipers to the nearest 0.01 mm. Weapon size was measured as the total length of the rostrum from the tip of the mandibles to the base of the head (RL). Body size measurements were total body length (BL, tip of mandibles through to end of elytra), pronotum width (PW), pronotum length (PL), elytra width (EW), and left elytra length (EL). A repeatability test was conducted by measuring 20 individual male *L. barbicornis* three times, and then calculating the fraction of the summed variance among individuals [29]. All body measurements had high levels of repeatability (0.99), and the variation between individuals was significantly higher than within individuals (RL: *F*_1,19_ = 323.5, *p* < 0.0001; PW: F_1,19_ = 257.2, *p* < 0.0001; PL: *F*_1,19_ = 487.3, *p* < 0.0001; EW: *F*_1,19_ = 276.8, *p* < 0.0001; EL: *F*_1,19_ = 282.6, *p* < 0.0001). Pronotum width was used in the following analyses as an overall measure of body size because this is the most common character used in the analysis of scaling relationships in other beetles [7,30,31]. Pronotum width was highly correlated with all other body size measurements (Pearson’s correlation coefficients compared to PW: Males BL = 0.988, PL = 0.983, EW = 0.991, EL = 0.987; Females BL = 0.986, PL = 0.969, EW = 0.990, EL = 0.982). 

### Trait trade-offs and compensation

To investigate the possibility of trade-offs and compensation between rostrum length and other traits, we collected 49 males at Matuku Reserve in March 2010. We attempted to collect representatives of all possible male body sizes, ranging from 18.5 to 82.1 mm in total body length (compared to a possible range of 15 to 90 mm). Each individual was frozen, and after measurement were stored in 70% Ethanol. We took body (PW) and weapon size (RL) measurements as above using Rok digital calipers to the nearest 0.01 mm and also measured the total length of the left antenna (AL), and the length of the left fore- (FTL) and hind-tibia (HTL). We measured a subset of 11 of these males three times and found high levels of repeatability (0.99) and the variation between individuals was significantly higher than within individuals (AL: *F*_1,10_ = 5467, *p* < 0.0001; FTL: *F*_1,10_ = 1550, *p* <0.0001; HTL: *F*_1,10_ = 2347, *p* <0.0001). The left hind wing was then dissected from each specimen and mounted on a slide with a drop of mounting agent (Microscopy Aquatex, Merck) and a cover slip. The width and length of each wing was measured under the microscope using an ocular micrometer (Leica M216 microscope). A subsample of 11 males were measured three times demonstrating high repeatability (0.99) of our measurements and the variation between individuals was significantly higher than within individuals (Wing 1: *F*_1, 10_ = 1861, *p* < 0.0001; Wing 2: *F*_1, 10_ = 4457, *p* < 0.0001). Testes were carefully dissected from each individual and immediately measured on a slide using a microscale (Bioquip) under a dissecting microscope. Giraffe weevils have bilobed testes, so to simplify the analysis only the width and length of the left lobe of the left testis was used. The average of the width and length of this lobe was taken as a measure of overall testis size (calculated as the (width + length)/2). A paired t-test showed no difference in the mean size of the right and left lobe (t = 1.68, df = 47, p = 0.10). We unfortunately did not conduct a repeatability test on these measurements, but as a proxy for measurement accuracy the Pearson’s correlation coefficient was calculated between mean size of left and right testis lobe (r = 0.93). We also ran the analyses using the width and length of the left lobe separately to check for any differences, but found the same result as using the mean; hence only data from the mean is presented here (but see Figure S1). 

### Statistical analysis

All statistical analyses were conducted in R 2.15 [32]. We first characterised the shape and slope of the scaling relationship for male and female weevils using individuals measured at Matuku Reserve. To analyse the shape of the scaling relationship between rostrum length and pronotum width the steps outlined in Knell [5] were followed. Six models were compared: (a) simple linear regression, (b) quadratic regression, (c) non-linear regression (logistic, four-parameter logistic, and Weibull growth function), and (d) breakpoint models using Akaike’s Information Criteria (AIC) and Bayesian Information Criteria (BIC) to determine the model which best described the data. After selecting the best model, AIC was used to compare models with different variance structures to check and account for heteroskedasticity. The breakpoint model, analysed using the *segmented* package in R [33,34], is useful when the relationship between trait and body size is best described by two straight lines rather than a single straight line or a curve [5,30]. Since Huxley [22] allometric relationships have been described using log-transformed data to enable easier fitting of an exponential model using linear regression. However, several recent publications have highlighted important issues with transforming raw data in these analyses, and modern techniques using non-linear regression makes log transformation no longer necessary [35-37]. To accurately describe the scaling relationship of *L. barbicornis* raw data was used, but the same models were also conducted using natural log transformation of both variables and it was found that very similar model selection applied (Table S1). 

The difference in the scaling relationship of rostrum length and body size (pronotum width) between males and females was analysed using the R package *smatr* Version 3.2.3 [38,39]. This package was developed specifically for analysing allometries, and uses type II regression techniques such as standardised major axis regression (SMA) to test whether a slope deviates from isometry (*b* < or > 1), or whether two slopes are significantly different from each other. This is a more appropriate technique than type I linear regression when the goal is to estimate the line of best fit from a bivariate data set rather than predicting the value of one variable from another because linear regression underestimates the line of best fit and could therefore lead to an incorrect decision as to whether two variables are isometric [38]. Although we have pointed out that issues are currently debated regarding the use of log transformation in allometry [35-37,40], there are currently no type II regression techniques available using raw data to test for analysing non-linear allometries. Therefore to enable comparison between the sexes data was log-transformed to test for positive allometry. We first tested for a deviation from isometry by fitting SMAs to male and female scaling relationships and then tested for a correlation between the standardized major and minor axes under an assumed slope of 1 [38]. A significant correlation (r) suggests that the true slope is not equal to 1. We also tested for a common slope between males and females using a test in *smatr* analogous to ANCOVA, but we used the slopes estimated by SMA rather than least squares regression [39]. 

The relative size of testes, wings, antennae, and legs were compared to weapon size (rostrum length) using residual analysis [13]. Firstly relative rostrum size was calculated by taking the residuals from a Weibull growth function analysis of rostrum length against body size (pronotum width) using untransformed data. Then, the relative size of each subsequent trait was determined by calculating the residuals from the most appropriate regression model of that untransformed trait size against untransformed pronotum width. To choose the best model to describe the scaling relationship between each trait and pronotum width, we compared the same six models (linear, quadratic, breakpoint, logistic, four-parameter logistic, and Weibull growth function) that were used to describe the weapon scaling relationship, and used AIC to choose the model that best fit the data (Table 1). Linear regression models were then conducted using relative trait size as the response variable, and relative rostrum length as the explanatory variable. A significant negative correlation indicates that males with relatively longer rostrums are trading off against other traits such as testes and wings. A positive correlation suggests that males are compensating for the increasing cost of bearing the weapon by increasing the size of the other trait. 

**Table 1:** Models fitted to describe the scaling relationship of untransformed trait size and pronotum width of male *Lasiorhynchus barbicornis*.

Trait	Model	AIC	ΔAIC
**Mean testis size**	**Linear**	**-24.24**	**0**
	Breakpoint	-22.72	1.52
	Quadratic	-22.3	1.94
	Logistic*	NA	NA
	Four-parameter logistic*	NA	NA
	Weibull growth function*	NA	NA
**Wing length**	**Breakpoint**	**104.97**	**0**
	Quadratic	105.26	0.29
	Logistic	105.36	0.39
	Four-parameter logistic	106.66	1.69
	Weibull growth function	106.75	1.78
	Linear	123.32	18.35
**Wing width**	**Quadratic**	**31.56**	**0**
	Logistic	32.01	0.45
	Breakpoint	33.64	2.08
	Weibull	33.82	2.26
	Linear	50.73	19.17
	Four-parameter logistic*	NA	NA
**Fore-tibia length**	**Breakpoint**	**20.07**	**0**
	Logistic	20.46	0.39
	Quadratic	21.48	1.41
	Four-parameter logistic	21.88	1.81
	Weibull growth function	21.95	1.88
	Linear	28.29	8.22
**Hind-tibia length**	**Four-parameter logistic**	**21.87**	**0**
	Breakpoint	24.18	2.31
	Weibull growth function	35.96	14.09
	Logistic	37.62	15.75
	Quadratic	40.09	18.22
	Linear	52.11	30.24
**Antenna length**	**Breakpoint**	**116.72**	**0**
	Four-parameter logistic	118.72	2
	Weibull growth function	118.78	2.06
	Logistic	120.33	3.61
	Quadratic	122.86	6.14
	Linear	138.85	22.13

* Due to model requirements, we were unable to fit non-linear models to these traits.

### Ethical Statement

The weevils used in this research were all collected from or measured at Matuku Reserve with permission from the Royal Forest and Bird Protection Society of New Zealand. 

## Results

### Shape and slope of scaling relationship

**Figure 2:**
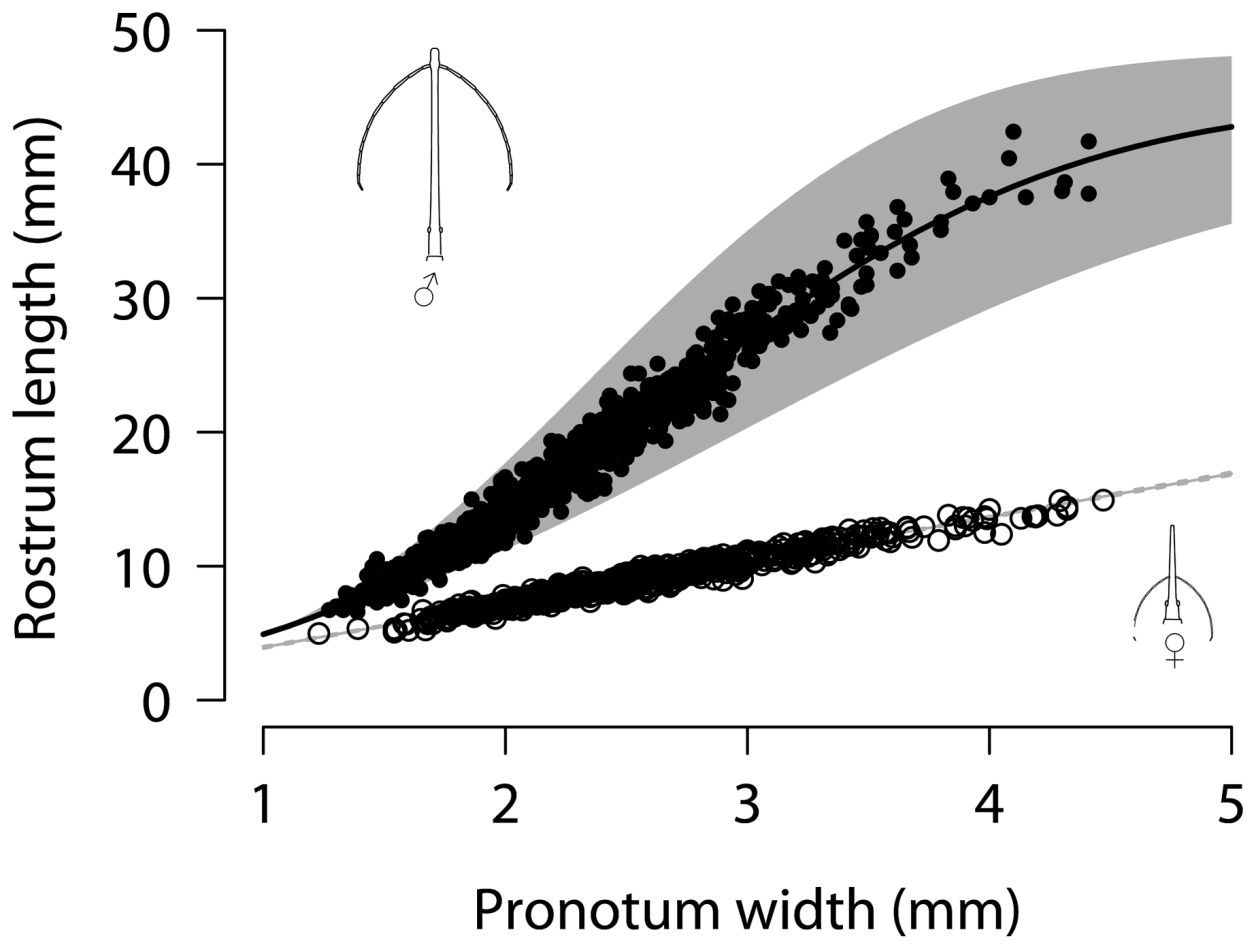
The allometry of rostrum length for male and female *Lasiorhynchus barbicornis*. The line of best fit for each sex is fitted on the data, showing a Weibull growth curve for males (closed circles), and a simple linear regression line for females (open circles). The grey shaded area around each set of points is the 95% confidence intervals for each model (seen as a grey line for females due to CI’s being very tight). Inset drawings show the sexual dimorphism in rostrum morphology (drawings by Vivian Ward).

To analyse the scaling relationship of female *L. barbicornis* a scatterplot of rostrum length against pronotum width was made to visually inspect the relationship (Figure 2). There was a clear linear relationship between these two traits, with no obvious deviation from a straight line. As expected, a simple linear regression model showed a very strong correlation between female rostrum length and body size (R^2^ = 0.96, rostrum length = 0.69 + 3.24x pronotum width, *p* <0.001). Following the parsimonious approach of Knell [5] we decided that it was not necessary to go further with this analysis, and it was concluded that the shape of the female scaling relationship is best described by a continuous, straight line. This conclusion is also supported by the extremely tight 95% confidence intervals for the slope estimate (Figure 2, 95% CI = 3.19 - 3.3). 

An initial inspection of a scatterplot of untransformed rostrum length (weapon size) against pronotum width (body size) for male *L. barbicornis* showed a continuous relationship between the two variables, but there appeared to be a decrease in relative rostrum length for the largest males (Figure 2). The slope appears to decrease at a pronotum width of approximately 3.8 mm. The scaling relationship was characterised by comparing a range of linear and non-linear models using AIC calculated as [2x (-log likelihood) + 2x (no. parameters in the model)] to compare models. Models with the lowest AIC values best describe the relationship between the variables in question, and models with a difference in AIC scores of more than two are considered to be different [41]. Changes in BIC (Bayesian Information Criterion) were also used to compare models as this approach takes into account sample size with the equation [-2x ln (likelihood) + no. parameters in the model x log (sample size)] [42]. BIC therefore penalizes more complicated models when sample size is large, and in this case can be used to avoid type I errors and ensure that the choice of a non-linear model to describe scaling relationship is really a better fit than a simple linear model.

**Figure 3:**
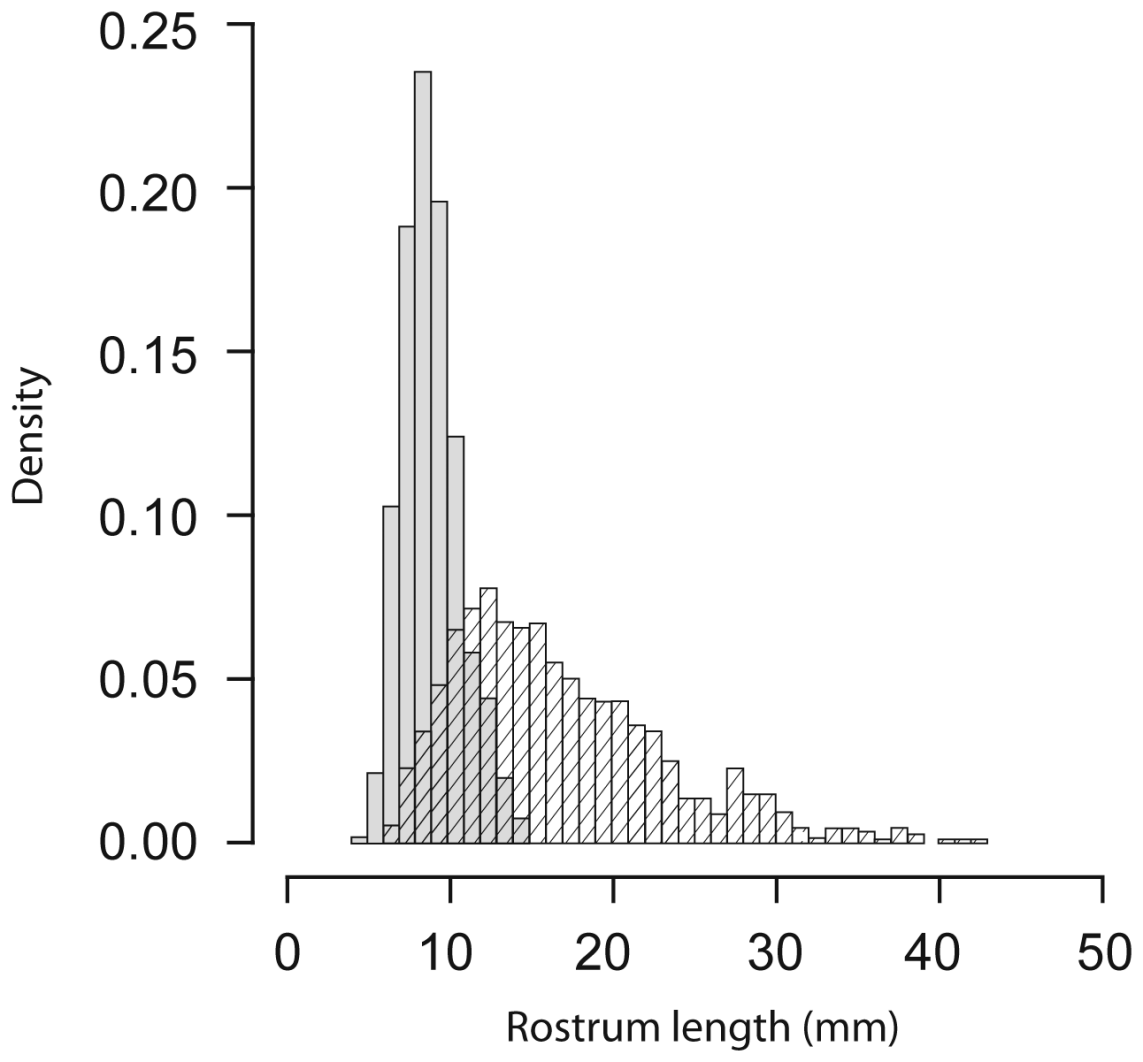
Frequency distribution of rostrum length in *Lasiorhynchus barbicornis*. Male (hatched bars, n = 987) rostrum length is right skewed but unimodal, while the rostrum length of females (shaded bars) is normally distributed. Frequencies are plotted as probability densities to allow for comparison between the two uneven sample sizes.

The allometric relationship between rostrum length and pronotum width was best described by a sigmoidal model (i.e. Weibull growth function), although the four-parameter logistic model showed very little difference in AIC and BIC weight (Table 2, Figure 2). Both models show that the upper asymptote is due to the decrease in relative rostrum size of a few very large males, although the four-parameter model also suggests some evidence of a lower asymptote. Overall, the deviation from linearity is slight, but the fit of both of these sigmoidal models were considerably better than a simple linear model (Weibull: ΔAIC = 98.9, ΔBIC = 89.1, Four-parameter logistic: ΔAIC = 98.1, ΔBIC = 88.4). The subtle differences in R^2^ between each model tested further emphasises that the deviation from linearity is slight and due to a small number of very large males. The frequency distribution of rostrum length for both males and females shows a unimodal distribution, but is highly right-skewed for males (Figure 3). 

**Table 2:** Models fitted to describe the scaling relationship of untransformed rostrum length and pronotum width of male *Lasiorhynchus barbicornis*.

Model	AIC	ΔAIC	BIC	ΔBIC	R^2^	Model Parameters
**Weibull growth function**	**3019.6**	**0**	**3044.1**	**0**	**0.971**	**a (asymptote) = 44.63**
						**drop (asymptote - y intercept) = 41.94**
						**lrc (ln rate constant) = -2.91**
						**power (power x is raised to) = 2.51**
Four-parameter logistic	3020.4	0.8	3044.8	0.7	0.971	a (lower asymptote) = -2.72
						b (upper asymptote) = 46.61
						c (scale) = 2.62
						d (x value for inflection point) = 0.92
Logistic model	3044.8	25.2	3064.4	20.3	0.970	a (asymptote) = 74.11
						b (mid value of x when y is a/2) = 1.36
						c (scale) = 0.46
Breakpoint model	3067.7	48.1	3092.2	48.1	0.969	intercept = -10.56
						slope left = 12.38
						breakpoint = 4.08
						slope right = -15.58
Linear model	3118.5	98.9	3133.2	89.1	0.968	a (intercept) = -10.26
						b (slope) = 12.23
Quadratic model	3120.2	100.6	3139.8	95.7	0.968	a (intercept) = -10.54
						b1 (first slope) = 12.47
						b2 (second slope) = -0.05

Note that the ΔAIC and ΔBIC were always calculated between the best model (in bold) and each subsequent model, arranged by increasing values of AIC and BIC.

To enable the comparison of the scaling relationship between the sexes and to test for deviation from isometry the slopes for each sex were treated as a straight line using log-transformed data. First, the allometric slope for males was calculated using all specimens, but to determine the effect of the decreasing slope for the largest males the slope was also calculated without these largest males. As the Weibull growth curve described earlier predicted an asymptote at a greater rostrum length than we observed (44.63 mm, max. observed was 42.45 mm), the allometric slope was calculated for males that had a pronotum width smaller than 4.08 mm, which was the predicted change in slope in the breakpoint model. Only six males (from 987) were above the predicted breakpoint, so the slope for these largest males was not calculated. The relationship between rostrum length and body size was steeply positively allometric for males, when including all males and when excluding males beyond the breakpoint, as indicated by the slope values which both show significant deviation from isometry (Table 3). The allometric slope is analogous to the power function in the Weibull growth curves (Table 2), however because data was logged for the SMA analysis the two values are not directly compared (but see Table S1). Females showed weak but significant negative allometry according to the test for deviation from isometry (Table 3). Using a likelihood ratio test compared to a chi-squared distribution it was found that the male and female slopes were significantly different from each other (Table 3). 

**Table 3:** Scaling relationship between body and rostrum size for males and females at Matuku Reserve.

	n	Slope (*b*)	lower CI	upper CI	r	χ^2^
Males (all)	987	1.65	1.63	1.67	0.94***	1362***
Males (ex. large males)	981	1.66	1.65	1.68	0.95***	1368***
Females	653	0.95	0.93	0.97	-0.21***	

Males above the predicted breakpoint were removed and the slope reanalysed without the largest males. The allometric slope is the standardised major axis slope (±95% confidence interval) of log rostrum length against log pronotum width. Slopes (b) significantly greater than 1 indicate a positive allometry, and *b* less than 1 indicates negative allometry. The r correlation statistic tests for deviation from isometry. The likelihood ratio (χ^2^) compares whether male and female slopes significantly differ from each other. *** indicates p <0.0001.

### Trade-offs and compensation

**Figure 4:**
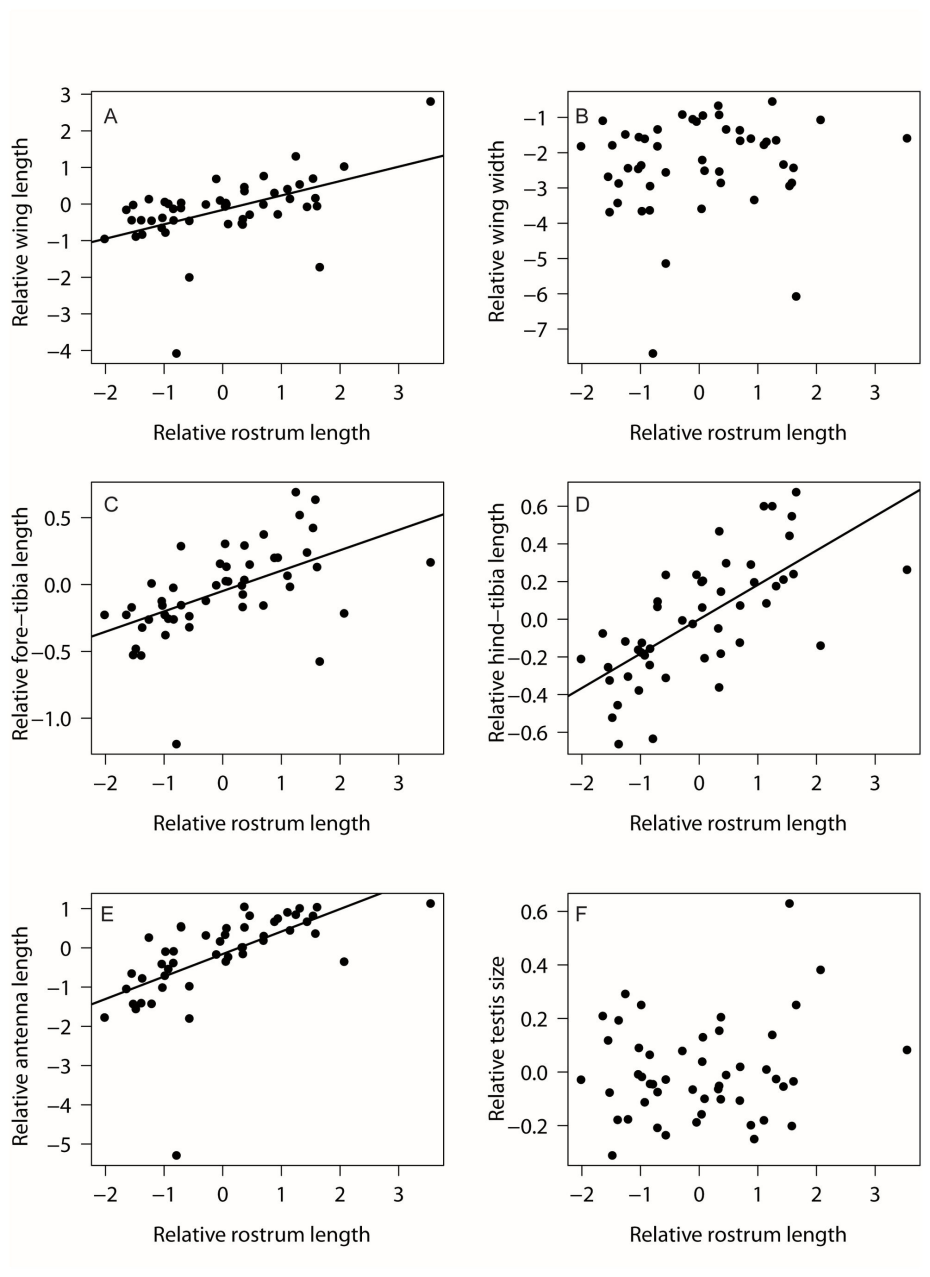
Relative trait size in relation to relative rostrum length for male *Lasiorhynchus barbicornis*. Relative trait size was calculated by taking the residuals of (A) wing length, (B) wing width, (C) fore-tibia length, (D) hind-tibia length, (E) antenna length, and (F) average of testis width and length from the best fit regression model of the trait against pronotum width. Linear regression line plotted for significant (*p* < 0.05) correlations only.

The residual analysis showed that relative wing length positively correlated with relative rostrum length, showing that males with the largest rostrums invest disproportionately more into wing length than males with smaller rostrums (Figure 4A; *b* = 0.394, df = 47, *p* <0.0001, R^2^ = 0.25). However, relative wing width did not significantly correlate with relative rostrum length (Figure 4B; *b* = 0.142, df = 47, *p* = 0.399, R^2^ = 0.02). Fore-tibia (Figure 4C; *b* = 0.152, df = 47, *p* <0.0001, R^2^ = 0.27), hind-tibia (Figure 4D; *b* = 0.183, df = 47, *p* <0.0001, R^2^ = 0.46), and antennae length (Figure 4E; *b* = 0.573, df = 47, *p* <0.0001, R^2^ = 0.37) showed positive correlations with relative rostrum length. Relative testis size did not correlate with relative rostrum length, suggesting that there is no trade-off or compensation between testes and rostrum size (Figure 4F; *b* = 0.024, df = 47, *p* = 0.28, R^2^ = 0.03).

## Discussion

### Shape of scaling relationship

The analysis of the scaling relationship between rostrum and body size showed a high level of dimorphism in the shape and steepness of the allometric slope between the sexes. The steep positive allometry displayed by males indicates that larger males are allocating a disproportionate amount of resources into rostrum length than smaller males. The presence of a positive allometry when studying an exaggerated trait in males (particularly when coupled with behavioural observations) is generally used as evidence that the trait is under sexual selection, and therefore demonstrates the importance of the trait as a weapon or ornament used during mate acquisition [3]. However, a recent review highlighted that not all sexually selected traits show positive allometries when scaled against body size, and not all traits showing positive allometries are under sexual selection [4]. An historic fascination with spectacularly exaggerated traits has led to the incorrect conclusion that all sexually selected traits exhibit positive allometry, while in fact this is not a universal trend across all taxa. When positive allometries are observed for an exaggerated trait, this suggests that (1) there is an overall selective advantage for larger males that leads to an increase in relative trait size (i.e. increasing trait size increases mating success more for larger males than small males), and (2) that there is corresponding selection for small males to invest relatively less in weapon size (i.e. small males cannot wield weapons effectively so invest in alternative mating tactics) [4,43]. Caution must therefore be taken, and it is important to couple this morphological data with behavioural observations that determine the significant role of the rostrum in male-male competition. Behavioural experiments determining whether males with relatively long rostrums have a competitive advantage during contests with other males will enable us to have a clearer understanding of how sexual selection might have shaped rostrum evolution in *L. barbicornis.*

Female rostrum length showed a linear relationship with body size, whereas males showed a clear tapering of the slope for the largest males, best explained by the Weibull growth function model (closely followed by the four-parameter logistic model) showing a sigmoidal relationship between weapon and body size. Male dimorphism is particularly common in highly size variable species that display weaponry, and although dimorphisms can be difficult to identify, modern techniques such as those outlined in Knell [5] that were followed in this study, have allowed these analyses to become easier. Despite this there was no evidence of dimorphism in weapon expression, rather rostrum length scaled steeply with body size until the curve starts to asymptote for the very largest males. This relationship suggests a physiological constraint on weapon size due to resource limitation in a similar pattern seen in other armed insects [7,21,44]. Furthermore, a frequency plot of rostrum length showed no evidence of bimodality. In *L. barbicornis* males of all sizes, despite extensive size variation, possess elongated rostra, and future studies will show how these are used across the spectrum of male sizes.

Asymptotes in weapon size in larger males are likely in species that have a relatively high investment into weapon size relative to body size [21]. For example, within the lucanid beetles, those species that have steep positive scaling relationships between mandible and body size show a higher level of constraint for the largest males than in those species that do not invest substantially into mandible size [21]. The declining investment into weaponry for the largest males in holometabolous insects such as beetles reflects the environment that these structures develop in, where most of the growth of the discs that will become adult appendages occurs after feeding has ceased, therefore reflecting the closed system in which they develop [19,20]. The growth of large exaggerated structures such as the rostrum in the giraffe weevil is therefore expected to be limited by available resources, which at the same time must be shared between all other developing adult structures. However, because these data are correlational we cannot rule out other possible reasons for a decrease in relative rostrum length such as limitations due to increasing energy requirements for wielding increasingly large weapons [21]. Another possible explanation for the decrease in relative rostrum length could be due to a diminishing requirement for large males to invest increasingly into larger rostra. Very large males are relatively rare in the population (Figure 3) and are therefore unlikely to directly compete with similarly large males on a regular basis. These large males are therefore likely to have the competitive advantage over the majority of males present in an aggregation and may not be under strong selection to invest further into rostrum length like their smaller counterparts. While the scaling relationship was best described by sigmoidal models, we recognise that the deviation from linearity in the model is subtle, and that a limitation in rostrum length is only occurring for the very largest males which are a scarce but important component of the population. Consequently, the scaling relationship for male giraffe weevils is a continuous and mostly linear relationship, with a decrease in slope for the very largest males. 

### Trade-offs and compensation

There was no evidence of a trade-off demonstrated by negative phenotypic correlation between rostrum size and any of the traits measured in males of the giraffe weevil. Among other species, previous studies investigating the potential for trade-offs between secondary sexual traits and other appendages have found mixed results. Some species show evidence of a trade-off, and others do not, demonstrating that there is no universal pattern across those taxa that display exaggerated traits. In the past, studies on trade-offs mostly focused on traits that were developing in close proximity to the exaggerated trait, and have found a tight link between the ecology of the species and the presence and location of these traits [13,45]. Trade-offs between adjacent developing traits are expected because the developing tissues mostly use a local pool of resources available for growth [13]. However many correlational [46-48] and experimental studies manipulating weapon size have identified trade-offs with disparate traits such as testes [25,49-51]. These patterns are not universal, possibly because trade-offs of this nature are only apparent when environmental conditions are stressful (i.e. during food shortages) [25,52]. Furthermore, by limiting the analysis to phenotypic correlations, it is not possible to determine whether underlying trade-offs are obscured by variation in individual quality due to genetic differences in the ability of individuals to acquire resources [53,54]. 

There was no correlation between relative testis size and rostrum length, indicating a lack of evidence for the predicted trade-off between rostrum length and testis size. The relative investment in weapon and testis size reflects an overall trade-off in traits that increase the likelihood of acquiring mates versus a trait that increases fertilisation success [25]. Giraffe weevil males are highly likely to experience sperm competition because females are highly promiscuous and will often mate with several males prior to oviposition (CJ Painting unpublished data). Males with the highest sperm competition risk are expected to be under selection for adaptations that reduce this risk, such as by increasing testis or ejaculate size [55]. In some species (e.g. Atlantic salmon, dung beetles, etc.) small males have been found to increase the relative investment into testis, presumably due to selection to compensate for an increase in sperm competition and a decrease in mating opportunities [48,56-59]. However, not all species with a small male morph increase relative testis size, perhaps due to variation in the cost of gaining mates in comparison to other species (e.g. [60]). One further possibility is that rather than increase testis size, small males instead have larger ejaculates per copulation because of a perceived disadvantage in mating opportunities [61]. 

The relative size of wings, antennae, and legs showed positive phenotypic correlations with relative rostrum length, indicating these traits increase in size as rostrum increases. This can be interpreted as males compensating for increased rostrum length (and possibly the increased cost of locomotion and fighting) by increasing the relative length of their hind wings, antennae, and legs. The development of exaggerated traits is repeatedly claimed to be costly, and can negatively affect the performance of other aspects of the individual not directly related to mate acquisition. If the production of an exaggerated trait negatively affects the performance of the individual (e.g. reduces flight ability), natural selection can operate to allow compensation of other physical or behavioural traits. Individuals that can reduce the cost of bearing the exaggerated trait will be at a selective advantage [15]. Compensation through integration of trait development can make it difficult to assess the cost of exaggerated trait production, especially if the individual is able to offset any reduction in performance, therefore complicating the ability to simply measure performance to imply the costliness of the trait [31]. We do not have data on flight in *L. barbicornis*, but intuitively one would expect that large males would have reduced flight capability due to the hindrance of bearing such long rostrums and additional weight. However, it is possible that by increasing the relative length of their hind wings, males have compensated for the cost of bearing a larger rostrum, and suffer little or no disadvantage in flight. Interestingly, although relative wing length showed the predicted correlation with rostrum length, wing width did not. The additional surface area resulting from a simple increase in relative wing length is perhaps enough to compensate for the burden of carrying a larger rostrum, such that it is not necessary for males to similarly increase relative wing width. This has been found to be the case in other species carrying large ornamentation, and highlights the need to assess the possibility of compensatory traits when studying the cost of sexually selected traits [15,31,62]. Compensation in wing size could be further analysed in the giraffe weevil by measuring flight behaviour between males across the size spectrum, and by using geometric morphometrics to assess variation in wing shape and its allometry in addition to wing length. Interpreting positive correlations as evidence of compensation and a lack of trade-offs in a study relying only on phenotypic correlations must be done with caution. Positive phenotypic correlations between traits can be observed even when an underlying negative genetic correlation due to a trade-off in resource allocation is expected [54]. These models have been better developed in the field of life history trade-offs, and have shown that there can be high levels of genetic variation in the ability of individuals to acquire resources from the environment, which can lead to differences in resource allocation [63]. Particularly when there is a large variation in how individuals acquire resources, a positive correlation between the phenotype of two size traits could simply be due to the ability of larger individuals to acquire resources, therefore having more available to allocate to both traits [54]. In a large-bodied species such as *L. barbicornis* which has a larval stage that lasts at least two years (CJ Painting unpublished data), breeding experiments to test the relative genetic variability in resource acquisition and allocation are prohibitive, and in this respect *L. barbicornis* may not make an ideal model to determine the genetic co-variance of these traits. While the lack of predicted trade-offs between traits, and the subsequent discovery of phenotypic correlation between traits could be interpreted as evidence for genetic correlation through developmental integration, we also recognise that these correlations are a complex reflection of differences in resource acquisition and allocation between individuals. 

Species with exaggerated traits that develop in a closed system, such as in beetles, are most likely to undergo competition with other developing traits, but are also likely to be under selective pressure to increase the size of traits that enable the exaggerated trait to be physically supported [13,14,20,49,64]. Genetic correlation due to developmental integration offers a possible explanation for the observation of increased investment in both fore- and hind-tibia size in relation to rostrum length. Males fight fiercely for access to females, using their rostrum and mandibles to push, pull and grapple with competing males (CJ Painting personal observations) [24]. The first part of this study showed that as overall body size increases, so does the relative investment into rostrum length, and therefore if larger, stronger legs are required to support the individual during fights it is not surprising that a correlated increased investment in leg size occurs through developmental integration [65]. Similar results have been found in other species, for example the fore-tibia in large, major males of *Onthophagus taurus* have been shown to be relatively larger than that of minor males, probably to compensate for the hindrance of bearing horns during digging in tunnels, and to support them during contests with rivals [14]. 

The observed increase in relative antenna length in relation to rostrum size is less likely to be explained by correlated selection to increase the success of large males. Instead it is more likely that the positive correlation between these two traits is due to direct interactions between cells during development because antennae grow directly from the surface of the rostrum. If the rostrum and antenna develop from the same module (i.e. same imaginal disc) any factor that causes variation in the growth of that module will be reflected in a positive correlation between the final adult traits [65]. Alternatively, positive correlations can occur even if the development of these traits is separate as signals between pathways can also generate covariation if the variation in one set of cells results in the communication of a chemical signal that affects the other cells in a similar way [65]. We must, therefore, acknowledge that this could be a possible explanation for the observed correlations between weapon size and wing and leg lengths. It is not known if the source of variation in body size (and rostrum length) in *L. barbicornis* is mostly due to genetic or environmental factors, but any variation that affects the size of one trait can manifest in covariation in another trait(s). The positive correlation between rostrum and antenna size in *L. barbicornis* reflects different developmental processes occurring compared to the production of horns in *Onthophagus* beetles, where horns and antennae share resources available for growth, but develop from different imaginal discs, resulting in trade-offs between the traits [13]. 

A principle tenet in sexual selection theory is that exaggerated traits are costly to produce and bear [1]. Giraffe weevil males can grow up to 90 mm long, and are the longest brentid weevil in the world, with their rostrum making up half of their total body length. With such a huge investment into rostrum length, it seems sensible to assume that this comes at the cost of other body parts. However, we did not find evidence that the production of the rostrum in this species inflicts costs on the relative size of any of the traits that we measured; rather large males actually increased their investment in wing, leg, and antenna length. We speculate that this is to compensate for the increased load during flight and fights, and in the case of antennae, this is perhaps due to developmental integration with rostrum size. Overall this study has shown that male giraffe weevils invest heavily into rostrum size which shows a steep positive relationship with body size, with the largest males under weapon-size constraint. This will therefore provide an important baseline for future studies on variation in body and weapon size investment. 

## Supporting Information

Figure S1 **Relative testis size in relation to relative rostrum length for male *Lasiorhynchus barbicornis*.** Relative testis size was calculated by taking the residuals of (A) testis width and (B) testis length from a linear regression model of the trait against pronotum width. A linear regression of relative testis size against relative rostrum length showed that there was no significant relationship between these traits (Testis width: *b* = 0.007, df = 47, *p* = 0.726, R^2^ = 0.003; testis length: *b* = 0.04, df = 47, *p* =0.104, R^2^ = 0.06).
(TIF)

Table S1 **Models fitted to describe the scaling relationship of natural log-transformed rostrum length and pronotum width of male *Lasiorhynchus barbicornis*.**
(DOCX)

## References

[1] Andersson M (1994) Sexual selection. Princeton, New Jersey: Princeton University Press.

[2] Darwin C (1871) The descent of man, and selection in relation to sex. London: Murray.

[3] Kodric-Brown A, Sibly RM, Brown JH (2006) The allometry of ornaments and weapons. Proc Natl Acad Sci U S A 103: 8733-8738. doi:10.1073/pnas.0602994103. PubMed: 16731616. 16731616 PMC1470970

[4] Bonduriansky R (2007) Sexual selection and allometry: A critical reappraisel of the evidence and ideas. Evolution 61: 838-849. doi:10.1111/j.1558-5646.2007.00081.x. PubMed: 17439616. 17439616

[5] Knell RJ (2009) On the analysis of non-linear allometries. Ecological Entomology 34: 1-11. doi:10.1111/j.1365-2311.2008.01022.x.

[6] Emlen DJ, Nijhout HF (2000) The development and evolution of exaggerated morphologies in insects. Annu Rev Entomol, 45: 661-708. PubMed: 10761593. 10761593 10.1146/annurev.ento.45.1.661

[7] Tomkins JL, Kotiaho JS, LeBas NR (2005) Matters of scale: Positive allometry and the evolution of male dimorphisms. Am Nat 165: 389-402. doi:10.1086/427732. PubMed: 15729668. 15729668

[8] Gross MR (1996) Alternative reproductive strategies and tactics: Diversity within sexes. Trends Ecol Evol 11: 92-98. doi:10.1016/0169-5347(96)81050-0. PubMed: 21237769. 21237769

[9] Oliveira RF, Taborsky M, Brockmann HJ (2008) Alternative Reproductive Tactics - An Integrative Approach. Cambridge: Cambridge University Press.

[10] Eberhard WG (1982) Beetle horn dimorphism - making the best of a bad lot. American Naturalist 119: 420-426. doi:10.1086/283920.

[11] Emlen DJ (1997) Alternative reproductive tactics and male-dimorphism in the horned beetle *Onthophagus* *acuminatus* (Coleoptera: Scarabaeidae). Behavioral Ecology and Sociobiology 41: 335-341. doi:10.1007/s002650050393.

[12] Moczek AP, Emlen DJ (2000) Male horn dimorphism in the scarab beetle, *Onthophagus* *taurus*: Do alternative reproductive tactics favour alternative phenotypes? Anim Behav 59: 459-466. doi:10.1006/anbe.1999.1342. PubMed: 10675268. 10675268

[13] Emlen DJ (2001) Costs and the diversification of exaggerated animal structures. Science 291: 1534-1536. doi:10.1126/science.1056607. PubMed: 11222856. 11222856

[14] Tomkins JL, Kotiaho JS, LeBas NR (2005) Phenotypic plasticity in the developmental integration of morphological trade-offs and secondary sexual trait compensation. Proc Biol Sci 272: 543-551. doi:10.1098/rspb.2004.2950. PubMed: 15799950. 15799950 PMC1578702

[15] Husak JF, Swallow JG (2011) Compensatory traits and the evolution of male ornaments. Behaviour 148: 1-29. doi:10.1163/000579510X541265.

[16] Ribak G, Swallow JG (2007) Free flight maneuvers of stalk-eyed flies: Do eye-stalks affect aerial turning behavior? J Comp Physiol A Neuroethol Sens Neural Behav Physiol 193: 1065-1079. doi:10.1007/s00359-007-0259-1. PubMed: 17710410. 17710410

[17] Swallow JG, Wilkinson GS, Marden JH (2000) Aerial performance of stalk-eyed flies that differ in eye span. J Comp Physiol B 170: 481-487. doi:10.1007/s003600000124. PubMed: 11128437. 11128437

[18] Emlen DJ, Allen CE (2003) Genotype to phenotype: Physiological control of trait size and scaling in insects. Integr Comp Biol 43: 617-634. doi:10.1093/icb/43.5.617. PubMed: 21680471. 21680471

[19] Nijhout HF, Wheeler DE (1996) Growth models of complex allometries in holometabolous insects. American Naturalist 148: 40-56. doi:10.1086/285910.

[20] Nijhout HF, Emlen DJ (1998) Developmental biology, evolution competition among body parts in the development and evolution of insect morphology. Proc Natl Acad Sci U S A 95: 3685-3689. doi:10.1073/pnas.95.7.3685. PubMed: 9520426. 9520426 PMC19896

[21] Knell RJ, Pomfret JC, Tomkins JL (2004) The limits of elaboration: Curved allometries reveal the constraints on mandible size in stag beetles. Proc Biol Sci 271: 523-528. doi:10.1098/rspb.2003.2641. PubMed: 15129963. 15129963 PMC1691621

[22] Huxley JS (1932) Problems of relative growth. London: Methuen.

[23] Kuschel G (2003) Fauna of New Zealand Number 45 Nemonychidae, Belidae, Brentidae (Insect: Coleoptera: Curculionoidea). Lincoln: Manaaki Whenua Press.

[24] Meads MJ (1976) Some Observations on *Lasiorhynchus* *barbicornis* (Brentidae: Coleoptera). New Zealand Entomologist 6: 171-176. doi:10.1080/00779962.1976.9722234.

[25] Simmons LW, Emlen DJ (2006) Evolutionary trade-off between weapons and testes. Proc Natl Acad Sci U S A 103: 16346-16351. doi:10.1073/pnas.0603474103. PubMed: 17053078. 17053078 PMC1637585

[26] Hardersen S, Macagno ALM, Sacchi R, Toni I (2011) Seasonal constraints on the mandible allometry of *Lucanus* *cervus* (Coleoptera: Lucanidae). European Journal of Entomology 108: 461-468.

[27] Hardersen S (2010) Seasonal variation of wing spot allometry in *Calopteryx* *splendens* . Ethology Ecology and Evolution 22: 365-373. doi:10.1080/03949370.2010.510042.

[28] Wong-Muñoz J, Córdoba-Aguilar A, del Castillo RC, Serrano-Meneses MA, Payne J (2011) Seasonal changes in body size, sexual size dimorphism and sex ratio in relation to mating system in an adult odonate community. Evolutionary Ecology 25: 59-75. doi:10.1007/s10682-010-9379-0.

[29] Whitlock MC, Schluter D (2009). The Analysis of Biological Data. Greenwood Village, Colorado. Roberts and Company Publishers.

[30] Eberhard WG, Gutierrez EE (1991) Male dimorphisms in beetles and earwigs and the question of developmental constraints. Evolution 45: 18-28. doi:10.2307/2409478. 28564069

[31] McCullough EL, Weingarden PR, Emlen DJ (2012) Costs of elaborate weapons in a rhinoceros beetle: how difficult is it to fly with a big horn? Behavioral Ecology 23: 1042-1048. doi:10.1093/beheco/ars069.

[32] R Core Team (2013) R: A Language and Environment for Statistical Computing. R Foundation for Statistical Computing Vienna, Austria Available: http://www.R-project.org.

[33] Muggeo VMR (2003) Estimating regression models with unknown break-points. Stat Med 22: 3055-3071. doi:10.1002/sim.1545. PubMed: 12973787. 12973787

[34] Muggeo VMR (2008) segmented: an R package to fit regression models with broken-line relationships. R NEWS 8: 20-25.

[35] Packard GC (2009) On the use of logarithmic transformations in allometric analyses. J Theor Biol 257: 515-518. doi:10.1016/j.jtbi.2008.10.016. PubMed: 19014956. 19014956

[36] Packard GC (2011) Unanticipated consequences of logarithmic transformation in bivariate allometry. J Comp Physiol B 181: 841-849. doi:10.1007/s00360-011-0565-3. PubMed: 21399952. 21399952

[37] Packard GC (2012) Is non-loglinear allometry a statistical artifact? Biological Journal of the Linnean Society 107: 764-773. doi:10.1111/j.1095-8312.2012.01995.x.

[38] Warton DI, Wright IJ, Falster DS, Westoby M (2006) Bivariate line-fitting methods for allometry. Biol Rev Camb Philos Soc 81: 259-291. doi:10.1086/506238. PubMed: 16573844. 16573844

[39] Warton DI, Duursma RA, Falster DS, Taskinen S (2012) SMATR 3 - an R package for estimation and inference about allometric lines. Methods in Ecology and Evolution 3: 257-259. doi:10.1111/j.2041-210X.2011.00153.x.

[40] Kerkhoff AJ, Enquist BJ (2009) Multiplicative by nature: Why logarithmic transformation is necessary in allometry. Journal of Theoretical Biology 257: 519-521. doi:10.1016/j.jtbi.2008.12.026.

[41] Akaike H (1973) Information theory as an extension of the maximum likelihood principle. In: Petrov BN Csaki F Second International Symposium on Information Theory. Budapest Akademiai Kiado.

[42] Schwarz G (1978) Estimating the dimension of a model. Annals of Statistics 6: 461-464. doi:10.1214/aos/1176344136.

[43] Bonduriansky R, Day T (2003) The evolution of static allometry in sexually selected traits. Evolution 57: 2450-2458. doi:10.1111/j.0014-3820.2003.tb01490.x. PubMed: 14686522. 14686522

[44] Zatz C, Werneck RM, Macias-Ordonez R, Machado G (2011) Alternative mating tactics in dimorphic males of the harvestman *Longiperna* *concolor* (Arachnida: Opiliones). Behavioral Ecology and Sociobiology 65: 995-1005. doi:10.1007/s00265-010-1103-0.

[45] Emlen DJ, Marangelo J, Ball B, Cunningham CW (2005) Diversity in the weapons of sexual selection: Horn evolution in the beetle genus *Onthophagus* (Coleoptera: Scarabaeidae). Evolution 59: 1060-1084. doi:10.1554/04-642. PubMed: 16136805. 16136805

[46] Pizzo A, Macagno ALM, Dusini S, Palestrini C (2012) Trade-off between horns and other functional traits in two *Onthophagus* species (Scarabaeidae, Coleoptera). Zoomorphology 131: 57-68. doi:10.1007/s00435-012-0148-1.

[47] Simmons LW, Emlen DJ, Tomkins JL (2007) Sperm competition games between sneaks and guards: A comparative analysis using dimorphic male beetles. Evolution 61: 2684-2692. doi:10.1111/j.1558-5646.2007.00243.x. PubMed: 17941836. 17941836

[48] Simmons LW, Tomkins JL, Hunt J (1999) Sperm competition games played by dimorphic male beetles. Proceedings of the Royal Society of London B. Biological Sciences 266: 145-150. doi:10.1098/rspb.1999.0614. PMC1690708 11007331

[49] Moczek AP, Nijhout HF (2004) Trade-offs during the development of primary and secondary sexual traits in a horned beetle. Am Nat 163: 184-191. doi:10.1086/381741. PubMed: 14970921. 14970921

[50] Okada K, Miyatake T (2009) Genetic correlations between weapons, body shape and fighting behaviour in the horned beetle *Gnatocerus* *cornutus* . Animal Behaviour 77: 1057-1065. doi:10.1016/j.anbehav.2009.01.008.

[51] Yamane T, Okada K, Nakayama S, Miyatake T (2010) Dispersal and ejaculatory strategies associated with exaggeration of weapon in an armed beetle. Proc Biol Sci 277: 1705-1710. doi:10.1098/rspb.2009.2017. PubMed: 20129986. 20129986 PMC2871848

[52] Messina FJ, Fry JD (2003) Environment-depedent reversal of a life history trade-off in the seed beetle *Callosobruchus* *maculatus* . J Evol Biol 16: 501-509. doi:10.1046/j.1420-9101.2003.00535.x. PubMed: 14635850. 14635850

[53] Metcalfe NB, Monaghan P (2003) Growth versus lifespan: perspectives from evolutionary ecology. Exp Gerontol 38: 935-940. doi:10.1016/S0531-5565(03)00159-1. PubMed: 12954479. 12954479

[54] van Noordwijk AJ, de Jong G (1986) Acquisition and Allocation of Resources: Their Influence on Variation in Life History Tactics. American Naturalist 128: 137-142. doi:10.1086/284547.

[55] Parker GA (1990) Sperm competition games: Sneaks and extra-pair copulations. Proceedings of the Royal Society of London B. Biological Sciences 242: 127-133. doi:10.1098/rspb.1990.0115.

[56] Gage MJG, Stockley P, Parker GA (1995) Effects of alternative male mating strategies on characteristics of sperm production in the Atlantic salmon (*Salmo* *salar*): Theoretical and empirical investigations. Philosophical Transactions of the Royal Society of London B: Biological Sciences 350: 391-399. doi:10.1098/rstb.1995.0173.

[57] Simmons LW, Tomkins JL, Alcock J (2000) Can minor males of Dawson's burrowing bee, *Amegilla* *dawsoni* (Hymenoptera: Anthophorini) compensate for reduced access to virgin females through sperm competition? Behavioral Ecology 11: 319-325. doi:10.1093/beheco/11.3.319.

[58] Stockley P, Purvis A (1993) Sperm competition in mammals: A comparative study of male roles and relative investment in sperm production. Functional Ecology 7: 560-570. doi:10.2307/2390132.

[59] Taborsky M (1998) Sperm competition in fish: `bourgeois' males and parasitic spawning. Trends Ecol Evol 13: 222-227. doi:10.1016/S0169-5347(97)01318-9. PubMed: 21238275. 21238275

[60] Munguia-Steyer R, Buzatto BA, Machado G (2012) Male dimorphism of a neotropical arachnid: Harem size, sneaker opportunities, and gonadal investment. Behavioral Ecology 23: 827-835. doi:10.1093/beheco/ars037.

[61] Kelly CD (2008) Sperm investment in relation to weapon size in a male trimorphic insect? Behav Ecol 19: 1018-1024. doi:10.1093/beheco/arn058.

[62] Oufiero CE, Garland T Jr. (2007) Evaluating performance costs of sexually selected traits. Functional Ecology 21: 676-689. doi:10.1111/j.1365-2435.2007.01259.x.

[63] Robinson MR, Beckerman AP (2013) Quantifying multivariate plasticity: genetic variation in resource acquisition drives plasticity in resource allocation to components of life history. Ecol Lett 16: 281-290. doi:10.1111/ele.12047. PubMed: 23301600. 23301600

[64] Klingenberg CP, Nijhout HF (1998) Competiion among growing organs and developmental control of morphological asymmetry. Proceedings of the Royal Society of London B. Biological Sciences 265: 1135-1139. doi:10.1098/rspb.1998.0409.

[65] Klingenberg CP (2004) Integration, modules and development: molecules to morphology to evolution. In: Pigliucci M Preston K Phenotypic integration: studying the ecology and evolution of complex phenotypes. New York: Oxford University Press pp. 213-230.

