# Supplementary figures and images for: Exaggerated Trait Allometry, Compensation and Trade-Offs in the New Zealand Giraffe Weevil (Lasiorhynchus barbicornis)
Source: PLoS One. 2013 Nov 27;8(11):e82467. doi: 10.1371/journal.pone.0082467 (PMC3842246; doi:10.1371/journal.pone.0082467)

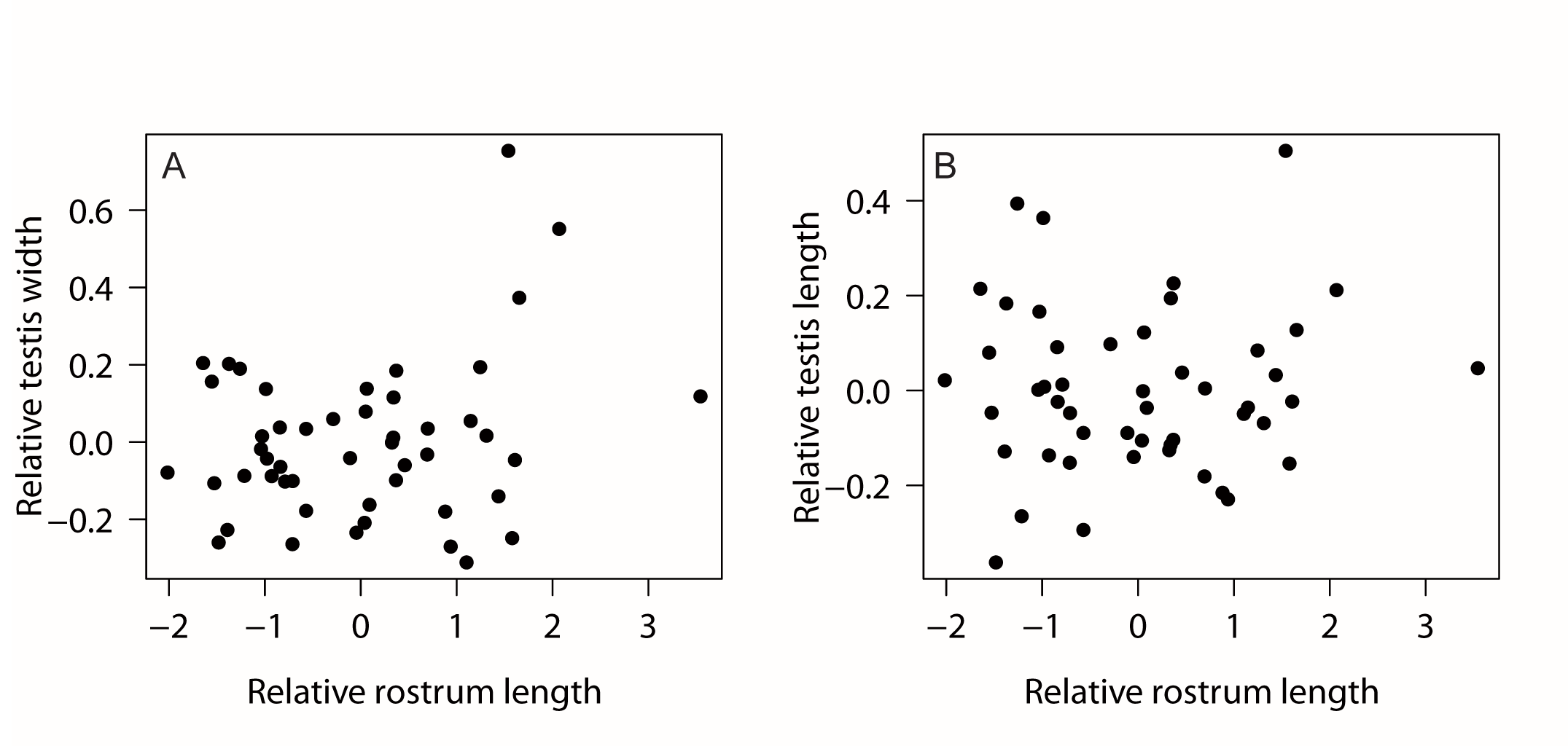

Supplement: Figure S1 — Relative testis size in relation to relative rostrum length for male Lasiorhynchus barbicornis. Relative testis size was calculated by taking the residuals of (A) testis width and (B) testis length from a linear regression model of the trait against pronotum width. A linear regression of relative testis size against relative rostrum length showed that there was no significant relationship between these traits (Testis width: b = 0.007, df = 47, p = 0.726, R2 = 0.003; testis length: b = 0.04, df = 47, p =0.104, R2 = 0.06). (TIF) [file pone.0082467.s001.tif]
