# Supplementary material for: Exaggerated Trait Allometry, Compensation and Trade-Offs in the New Zealand Giraffe Weevil (Lasiorhynchus barbicornis)
Source: PLoS One. 2013 Nov 27;8(11):e82467. doi: 10.1371/journal.pone.0082467 (PMC3842246; doi:10.1371/journal.pone.0082467)
Supplement: Table S1 — Models fitted to describe the scaling relationship of natural log-transformed rostrum length and pronotum width of male Lasiorhynchus barbicornis. (DOCX) [file pone.0082467.s002.docx]

**Table S1. Models fitted to describe the scaling relationship of natural log-transformed rostrum length and pronotum width of male *Lasiorhynchus barbicornis****.*

| Model | AIC | ΔAIC | BIC | ΔBIC | Model Parameters |
| --- | --- | --- | --- | --- | --- |
| **Weibull growth function** | **-2597.5** | **0** | **-2572.6** | **0** | **a (asymptote) = 4.11** |
|  |  |  |  |  | **drop (asymptote minus y intercept) = 2.35** |
|  |  |  |  |  | **lrc (ln rate constant) = -0.12** |
|  |  |  |  |  | **power (power x is raised to) = 1.77** |
| **Four parameter logistic** | **-2597.5** | **0** | **-2573.0** | **0.44** | **a (lower asymptote) = 1.15** |
|  |  |  |  |  | **b (upper asymptote) = 4.11** |
|  |  |  |  |  | **c (scale) = 0.69** |
|  |  |  |  |  | **d (x value for inflection point) = 0.42** |
| Breakpoint | -2588.9 | 8.6 | -2564.4 | 8.18 | intercept = 1.47 |
|  |  |  |  |  | slope left = 1.68 |
|  |  |  |  |  | breakpoint = 1.1 |
|  |  |  |  |  | slope right = -0.61 |
| Quadratic | -2556.3 | 41.2 | -2536.7 | 35.87 | intercept = 1.34 |
|  |  |  |  |  | slope 1 = 2.06 |
|  |  |  |  |  | slope 2 = -0.27 |
| Linear | -2483.4 | 114.1 | -2468.7 | 103.85 | intercept = 1.51 |
|  |  |  |  |  | slope = 1.62 |

Models are arranged by increasing values of AIC and BIC. Note that the ΔAIC and ΔBIC were always calculated between the best model (in bold) and each subsequent model.
